# Potential Surviving Effect of *Cleome droserifolia* Extract against Systemic *Staphylococcus aureus* Infection: Investigation of the Chemical Content of the Plant

**DOI:** 10.3390/antibiotics13050450

**Published:** 2024-05-15

**Authors:** Jawaher Alqahtani, Walaa A. Negm, Engy Elekhnawy, Ismail A. Hussein, Hassan Samy Hassan, Abdullah R. Alanzi, Ehssan Moglad, Rehab Ahmed, Sarah Ibrahim, Suzy A. El-Sherbeni

**Affiliations:** 1 Department of Pharmacognosy, College of Pharmacy, King Saud University, Riyadh 11495, Saudi Arabia; aralonazi@ksu.edu.sa; 2 Department of Pharmacognosy, Faculty of Pharmacy, Tanta University, Tanta 31527, Egypt; walaa.negm@pharm.tanta.edu.eg (W.A.N.); suzy.elsherbini@pharm.tanta.edu.eg (S.A.E.-S.); 3 Department of Pharmaceutical Microbiology, Faculty of Pharmacy, Tanta University, Tanta 31527, Egypt; 4 Department of Pharmacognosy and Medicinal Plants, Faculty of Pharmacy (Boys), Al-Azhar University, Cairo 11884, Egypt; ismaila.hussein@azhar.edu.eg; 5 Faculty of Pharmacy, Tanta University, Tanta 31527, Egypt; hasan30998367@pharm.tanta.edu.eg; 6 Department of Pharmaceutics, College of Pharmacy, Prince Sattam bin Abdulaziz University, P.O. Box 173, Alkharj 11942, Saudi Arabia; e.moglad@psau.edu.sa; 7 Department of Natural Products and Alternative Medicine, Faculty of Pharmacy, University of Tabuk, Tabuk 47713, Saudi Arabia; rahmed@ut.edu.sa; 8 Human Anatomy and Embryology Department, Faculty of Medicine, Tanta University, Tanta 31527, Egypt; sara.ibrahim@med.tanta.edu.eg

**Keywords:** antibiotic resistance, biofilm, systemic infection, LC-ESI-MS/MS, qRT-PCR, inflammatory markers

## Abstract

The increasing rates of morbidity and mortality owing to bacterial infections, particularly *Staphylococcus aureus* have necessitated finding solutions to face this issue. Thus, we elucidated the phytochemical constituents and antibacterial potential of *Cleome droserifolia* extract (CDE). Using LC-ESI-MS/MS, the main phytoconstituents of CDE were explored, which were kaempferol-3,7-*O*-bis-alpha-L-rhamnoside, isorhamnetin, cyanidin-3-glucoside, kaempferide, kaempferol-3-*O*-alpha-L-rhamnoside, caffeic acid, isoquercitrin, quinic acid, isocitrate, mannitol, apigenin, acacetin, and naringenin. The CDE exerted an antibacterial action on *S. aureus* isolates with minimum inhibitory concentrations ranging from 128 to 512 µg/mL. Also, CDE exhibited antibiofilm action using a crystal violet assay. A scanning electron microscope was employed to illuminate the effect of CDE on biofilm formation, and it considerably diminished *S. aureus* cell number in the biofilm. Moreover, qRT-PCR was performed to study the effect of CDE on biofilm gene expression (*cna*, *fnb*A, and *ica*A). The CDE revealed a downregulating effect on the studied biofilm genes in 43.48% of *S. aureus* isolates. Regarding the *in vivo* model, CDE significantly decreased the *S. aureus* burden in the liver and spleen of CDE-treated mice. Also, it significantly improved the mice’s survival and substantially decreased the inflammatory markers (interleukin one beta and interleukin six) in the studied tissues. Furthermore, CDE has improved the histology and tumor necrosis factor alpha immunohistochemistry in the liver and spleen of the CDE-treated group. Thus, CDE could be considered a promising candidate for future antimicrobial drug discovery studies.

## 1. Introduction

*Cleome droserifolia* (Forssk.) Delile Descr. is a small shrub that can grow to 60 cm in height. It grows naturally in Egyptian deserts like the Sinai Peninsula, stone soil, and rocky wadis. It became endangered due to extensive uprooting in Egypt’s Sinai and Eastern Deserts [1,2]. The genus *Cleome* (L.) DC. (Cleomacaea) [3] encompasses annual and perennial medicinal herbs or small shrubs [4,5,6,7]. *Cleome droserifolia*, known by local Bedouin people as Samwah, is employed in traditional medicine in Egypt as a hypoglycemic agent [8,9]. Different researchers analyzed the phytochemical content of *C. droserifolia*, exploring the existence of flavonoids [10], alkaloids, tannins, saponins, coumarins, catechins, sterols, glucosinolates, and terpenoids [1,9]. The essential oil profile of *C. droserifolia* was also studied, and the main compounds in the essential oil were (Z)-nerolidol and *α*-cadinol [11]. *C. droserifolia* exerted different biological effects as an antioxidant, antimicrobial [12], anticancer [13], allelopathic [11], and hypoglycaemic [14] properties.

*Staphylococcus aureus* is a common species of *Staphylococci* highly associated with multidrug resistance [15]. Such bacterial species can adapt to various environments and possess frequent virulence factors [16]. In addition, it is a common nosocomial pathogen that can trigger various diseases that range from mild severity to life-threatening ailments. The infections triggered by *S. aureus* are mild skin and soft tissue infections, bacteremia, osteomyelitis, endocarditis, and pneumonia [17].

*S. aureus* can exhibit resistance to antibiotics by various mechanisms, including decreasing the bacterial membrane permeability to the antibiotics, efflux, and excessive production of resistance enzymes like β-lactamases [18]. Multi-drug resistance (MDR) is a worldwide issue that has a deleterious effect on health care. *S. aureus* acquires resistance to antibiotics owing to persistent exposure to various antimicrobials. MDR *S. aureus* is resistant to multiple chemotherapeutic agents. Such MDR isolates have led to increasing global rates of mortality as well as morbidity in *S. aureus-*infected patients [19].

Biofilm is an important virulence factor of *S. aureus*, and it is defined as an extracellular complex structure that compromises a population of bacterial cells anchored to living or non-living surfaces [20]. The cells are surrounded by an extracellular polymer matrix formed by themselves as a protective tactic for bacterial survival to adapt to their surrounding environments [21].

Traditional antibiotics are excessively losing their potential to combat bacterial infections, particularly *S. aureus* [22]. Thus, novel treatment approaches should be elucidated to face such global concerns. Natural sources, like plants, are rich in many bioactive phytochemicals with various therapeutic activities [23]. Many studies have elucidated the potential antimicrobial action of plants and their bioactive constituents against viruses, fungi, and bacteria [24,25,26,27,28].

Here, we aimed to explore CDE’s potential antibacterial action *in vitro* and *in vivo*. Also, the phytoconstituents of this plant will be elucidated using liquid chromatography-electrospray ionization-tandem mass spectrometry (LC-ESI-MS/MS) to explore the active principles with different chemical entities which may have a role in developing new antibacterial agents against the MDR *S. aureus* isolates.

## 2. Results

### 2.1. Recognition of Different Phytochemical Contents of C. droserifolia by LC-ESI-MS/MS

It was revealed tentatively by this technique that *C. droserifolia* contains different phytochemical groups with variable biological effects. This plant contains dicarboxylic and tricarboxylic acids and their derivatives, hydroxy fatty acids, hydroxybenzoic acid derivatives, flavonols, flavones, flavanones, aurone O-glycosides, hydroxycinnamic acids, quinic acids and derivatives, flavonoid-3-*O*-glycosides, anthocyanidin-3-*O*-glycosides, anthocyanidin-5-*O*-glycosides, alkyl glucosinolate, methoxy phenols, and 4′-*O*-methylated flavonoids. The LC-ESI-MS/MS analysis disclosed that the predominant constituents are kaempferol-3,7-*O*-bis-alpha-L-rhamnoside, isorhamnetin, cyanidin-3-glucoside, 3, 5, 7-trihydroxy-4′-methoxyflavone (kaempferide), kaempferol-3-*O*-alpha-L-rhamnoside, caffeic acid, isoquercitrin, quinic acid, isocitrate, mannitol, apigenin, acacetin, and naringenin. Table 1 and Figure S1 in the Supplementary Materials demonstrate the tentatively recognized compounds supported by referenced data. The previously reported data revealed the presence of different phenolic compounds in *C. droserifolia*, such as kaempferol-3,7-dirhamnoside, isorharmnetin-3-*O*-gluco-7-*O*-rhamnoside, kaempferol-3-*O*-gluco-7-*O*-rhamnoside, quercetin-3-*O*-gluco-7-*O*-rhamnoside, kaempferol, artemitin [9], Isorhamnetin-3-*O-*β-d-glucoside, quercetin-3′-methoxy-3-*O*-(4″-acetyl rhamnoside)-7-*O*-α-rhamnoside, and kaempferol-4′-methoxy-3,7-dirhamnoside [29]. It was detected by RP-HPLC of the methanolic extract of the plant that the major phenolic compounds were benzoic acid, ellagic acid, rutin, o-coumaric acid, and naringenin. Moderate quantities of rosmarinic acid, p-hydroxybenzoic acid, resveratrol, kaempferol, quercetin, and ferulic acid were detected. The least abundant phenolic compounds were chlorogenic acid, caffeic acid, p-coumaric acid, syringic acid, and catechin [12].

### 2.2. Bacterial Isolates and Antibiotic Resistance

**Figure 1:**
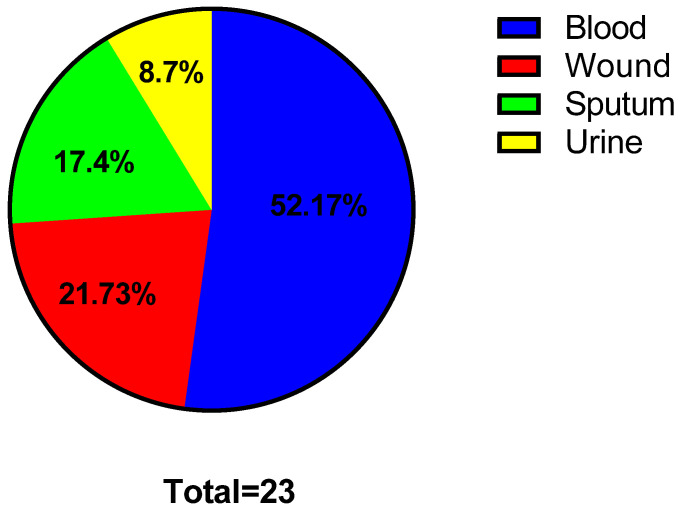
Pie chart revealing the percentages of the clinical specimens.

**Figure 2:**
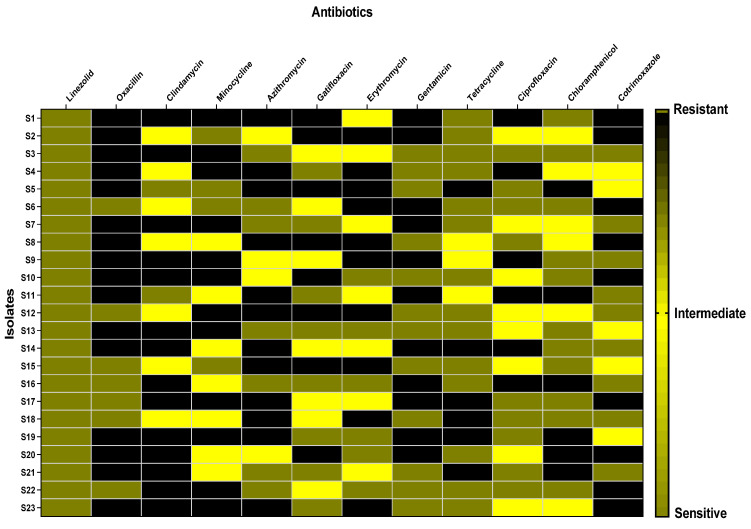
Heat map representing the susceptibility of *S. aureus* isolates to different antibiotics.

The clinical specimens from which the bacterial isolates were recovered include blood, wounds, sputum, and urine (Figure 1). The antibiotic susceptibility of the tested isolates is revealed in Figure 2.

### 2.3. Susceptibility of S. aureus to CDE

**Figure 3:**
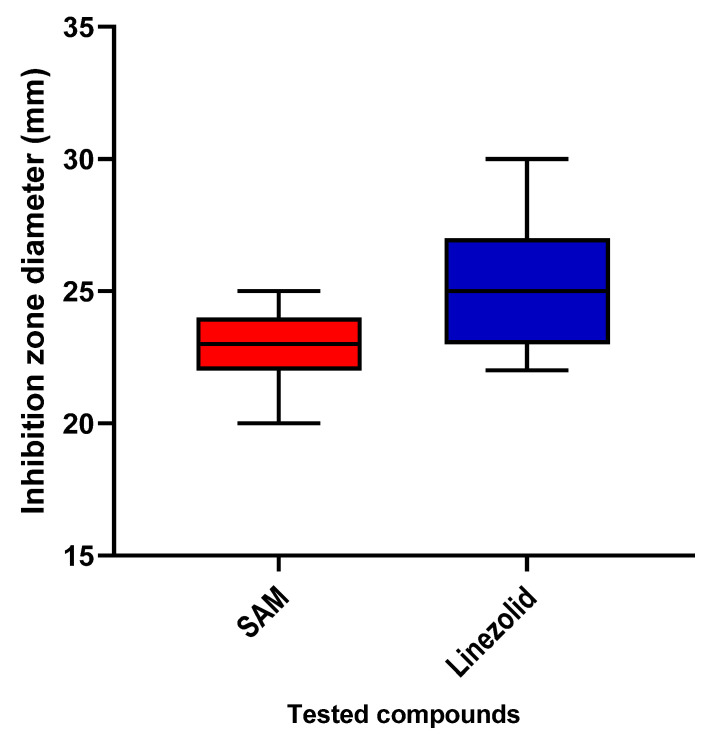
The inhibition zone diameters of CDE against the tested isolates.

The susceptibility of *S. aureus* to CDE was elucidated using the agar well diffusion method as a preliminary method to reveal whether CDE possesses antibacterial action (Figure 3 and Table S2). Then, the MICs were determined, as shown in Table S3.

### 2.4. Determination of Antibiofilm Potential by Crystal Violet Assay, SEM, and qRT-PCR

**Figure 4:**
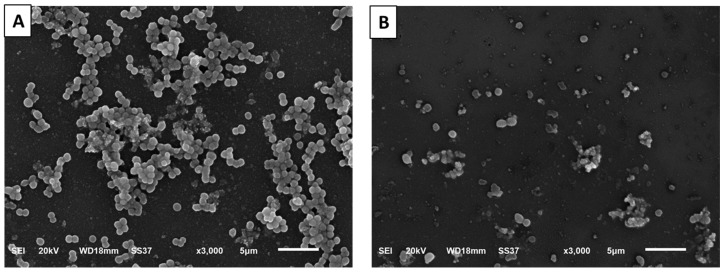
SEM micrograph revealing the morphology of *S. aureus* biofilm formed on the surfaces of cover glass: (**A**) without (untreated isolates) and (**B**) with CDE (treated isolates).

CDE revealed antibiofilm potential through the semiquantitative method, crystal violet (Table 2), as it decreased the percentage of strong and moderate biofilm-forming isolates from 73.91% to 30.43%. Then, SEM was employed to elucidate the effect of CDE on the morphology of the biofilm (Figure 4). CDE has significantly decreased the number of cells in the formed biofilm.

**Figure 5:**
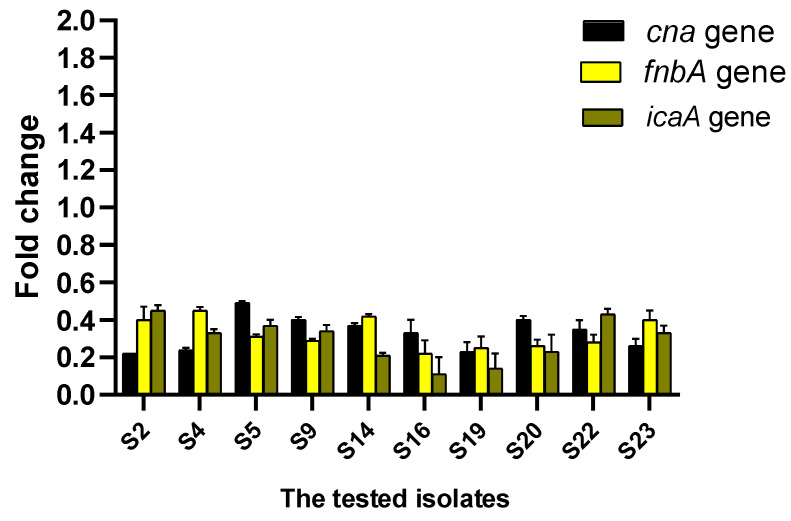
Impact of CDE on the gene expression levels of the biofilm.

qRT-PCR was utilized to reveal the potential of CDE on the expression level of biofilm genes. CDE was found to downregulate the biofilm-encoding genes in 43.48% of the isolates, as revealed in Figure 5.

### 2.5. In Vivo Infection Model in Mice

**Figure 6:**
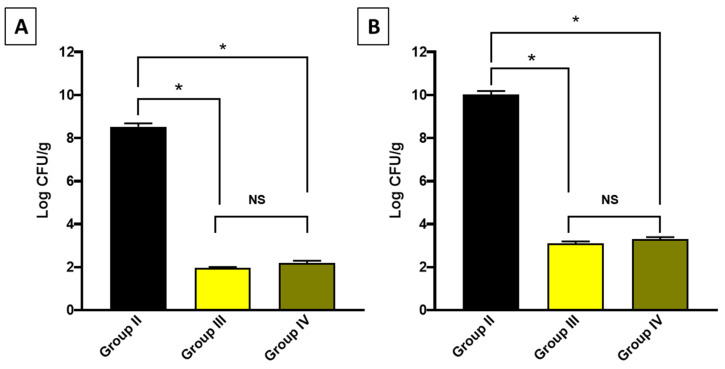
Bacterial burden in (**A**) the liver and (**B**) the spleen. The symbol (*) denotes a significant difference (*p* < 0.05). The abbreviation (NS) denotes a non-significant difference (*p* > 0.05).

A systemic infection was induced in mice, and the bacterial burden was detected in the liver and spleen in the experimental groups (Figure 6).

**Figure 7:**
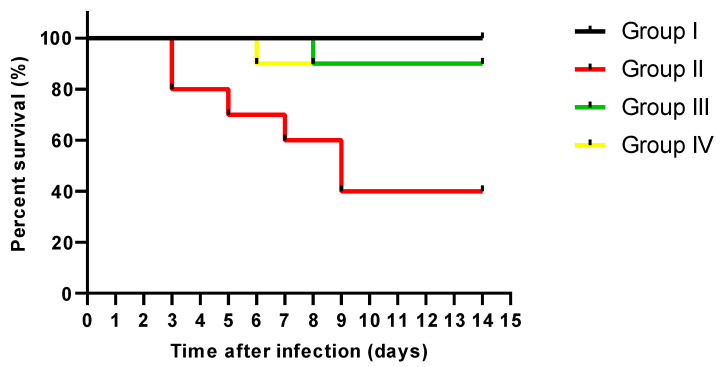
Survival curve constructed for the experimental groups.

The survival curve was constructed as shown in Figure 7. No mice died in group I; in group II, two died after three days, one after five days, and another after one week. Also, two mice died after nine days. Regarding groups III and IV, one mouse died on the eighth and sixth days.

### 2.6. Histopathological and Immunohistochemical Investigations

**Figure 8:**
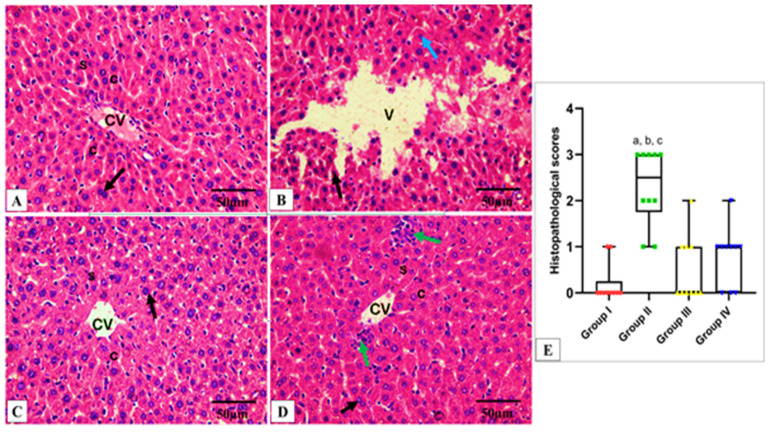
Light microscopic H&E-stained images of hepatic sections of adult albino rats of all studied groups. (**A**) Group I shows normal hepatic architecture in the form of hepatic cords (c) radiating from the central vein (CV) and separated by the hepatic sinusoids (S), with pericentral zone and midzone hepatocytes. Polyhedral hepatocytes appear with rounded vesicular nuclei and granular eosinophilic cytoplasm (black arrow) separated by sinusoids (S) lined with endothelial cells and Kupffer cells. (**B**) Group II shows disturbed hepatic architecture, compressed hepatic sinusoids, multiple pyknotic nuclei (black arrow), multiple karyolitic nuclei (blue arrow), and diffuse vacuolar degeneration of hepatocytes with multiple large vacuoles (V) as well as ballooned hepatocytes. (**C**) Group III shows organized hepatic cords (C) around the central vein, resembling the normal hepatic structure of Group I. (**D**) Group IV shows marked improvement and a regaining of the normal hepatic structure. However, with this enhancement, inflammatory cells infiltrate the pericentral zone (green arrow). (**E**) Histopathological score of the hepatic tissue cross sections in all studied groups. A box plot was used to express the data. The bottom of the plot represents 25%, the middle represents the median, and the top represents 75% of the data. Significant difference at *p* ≤ 0.05, where (a) in comparison with group I, (b) in comparison with group III, and (c) in comparison with group IV using the Kruskal–Wallis test followed by Dunn’s pairwise comparison post-hoc test. (H&E × 400, scale bar = 50 μm).

**Figure 9:**
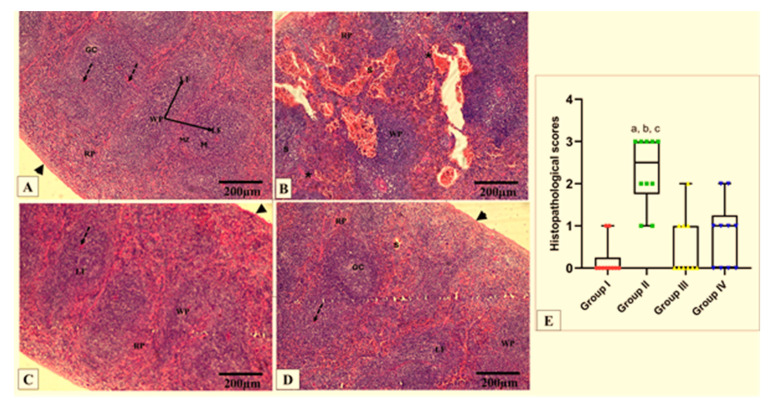
Photomicrographs of H&E-stained sections of spleen in all studied groups. (**A**) Group I shows the normal histological organization of white pulp (WP) and red pulp (RP) surrounded by a capsule (arrowhead). The WP presents the central arteriole (dashed arrow) and lymphoid follicles (LF), with germinal centers (GC) and mantle regions (M), surrounded by a loosely distributed marginal zone (MZ). The RP presents lymphocytes, trabeculae, and sinusoids. (**B**) Group II shows a loss of normal architecture, shrunken WP, and broadened RP. Note congested, dilated splenic sinuses (S) in the RP. Many cells in the WP appear vacuolated. Thick fibrous trabeculae are observed (asterisk). (**C**) Group III shows a nearly normal appearance of the WP and RP splenic architecture. (**D**) Group IV shows a nearly normal outline of splenic architecture, but congested splenic sinuses are also seen. (**E**) Histopathological score of the splenic tissue cross sections in all studied groups. A box plot was used to express the data. The bottom of the plot represents 25%, the middle represents the median, and the top represents 75% of the data. Significant difference at *p* ≤ 0.05 (a) in comparison with group I, (b) in comparison with group III, and (c) in comparison with group IV using the Kruskal–Wallis test followed by Dunn’s pairwise comparison post-hoc test. (H&E × 100, scale bar = 200 μm).

**Figure 10:**
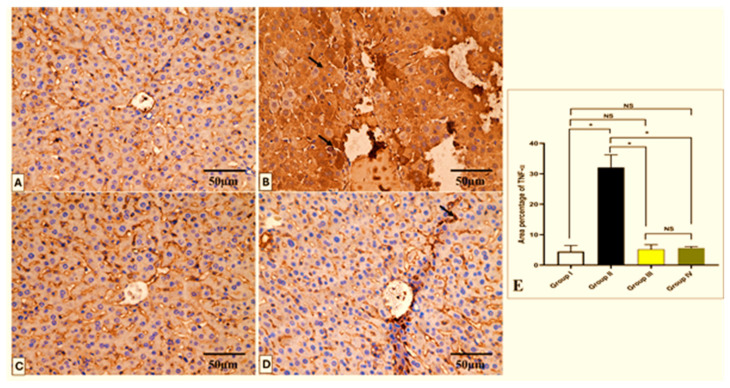
Light microscopic TNF-α-stained pictures of a liver section in all studied groups. (**A**) Group I shows a negative TNF-α expression within the hepatocytes’ cytoplasm. (**B**) Group II shows a strong positive expression of TNF-α, which appears as brownish cytoplasm in hepatocytes (arrows). (**C**) Group III shows a negative expression of TNF-α. (**D**) Group IV shows a mild positive expression of TNF-α in a few hepatocytes (arrow) and a negative expression in most of the cells. (**E**) The area percentage of TNF-α in each group. Mean ± SD was used to represent the data. One-way ANOVA was used for the statistical comparison, and Tukey’s post-hoc test was used for multiple comparisons. The single asterisk indicates a significant change, and the abbreviation NS denotes a non-significant change (*p* < 0.05). (TNF-α × 400, scale bar = 50 μm).

**Figure 11:**
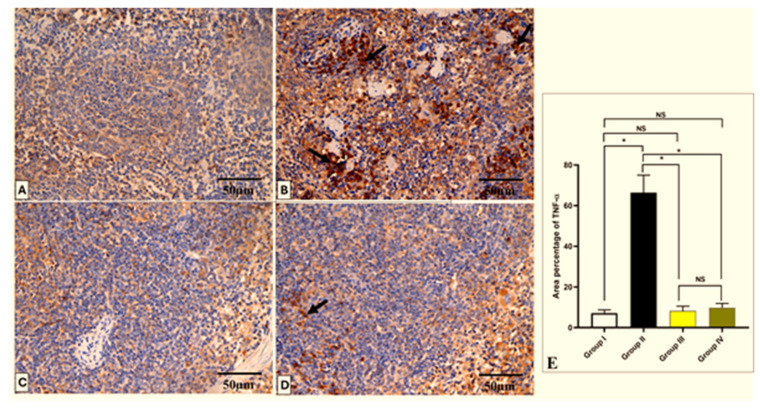
Light microscopic TNF-α-stained pictures of the spleen in all studied groups. (**A**) Group I shows a negative expression of TNF-α within the cytoplasm of the cells. (**B**) Group II shows a strong positive expression of TNF-α, which appears as brownish cytoplasm in most cells (arrows). (**C**) Group III shows a negative expression of TNF-α. (**D**) Group IV shows few TNF-α-positive cells (arrow) and a negative expression in most cells. (**E**) The area percentage of TNF-α in each group. Mean ± SD is used to represent the data. One-way ANOVA was used for the statistical comparison, and Tukey’s post-hoc test was used for multiple comparisons. The single asterisk indicates a significant change, and the abbreviation NS denotes a non-significant change (*p* < 0.05). (TNF-α × 400, scale bar = 50 μm).

The effect of CDE on the histological features of the liver and spleen is shown in Figure 8 and Figure 9. Also, the TNF-α immunohistochemical staining of the liver and spleen of the different experimental groups is shown in Figure 10 and Figure 11.

### 2.7. ELISA

Levels of IL-1β and IL-6 were detected in the liver and spleen tissues of the different groups (Table 3).

## 3. Materials and Methods

### 3.1. Collection, Drying, and Extraction of the Plant Material

The stems, flowers, and leaves of *C. droserifolia* shrubs were collected in March 2022 from the wadis around Sharm El-Sheikh and Dahab in South Sinai Governorate. The plant was dried and powdered to obtain 1.2 kg of dry weight. It was recognized by Prof. Dr. Hanafey Farouk Maswada, Professor of Plant Physiology, Agricultural Botany Department, Faculty of Agriculture, Tanta University, and depositing a voucher specimen (PG-A-00124) in the herbarium of the Department of Pharmacognosy, Faculty of Pharmacy, Tanta University. Methanol (Sigma Chemical Co., St. Louis, MO, USA) was used as an extracting solvent and was mixed with the powder. The extraction was performed thrice (4 L × 3 times), and then the concentration of the solvent was performed by a rotary evaporator under vacuum to obtain 45.3 g of dry residue from the plant’s extract.

### 3.2. Exploration of the Plant’s Phytoconstituents by LC-ESI-MS/MS

Different compounds in the extract were identified by the Proteomics and Metabolomics Unit, Children’s Cancer Hospital (57357), Basic Research Department, Cairo, Egypt. The crude extract was reconstituted in DI-Water:Methanol:Acetonitrile—50:25:25, and HPLC separation was accomplished by using in-line filter disks (0.5 µm × 3.0 mm, Phenomenex^®^, Torrance, CA, USA) and X select HSS T3 (2.5 µm, 2.1 × 150 mm, Waters^®^, Milford, MA, USA, 40 °C). The first is a pre-column, and the second is an analytical column. The mobile phases were composed of mobile phase A, which is composed of 5 mM ammonium formate buffer pH 8 with 1% methanol. Mobile phase B is 100% acetonitrile. Isocratic elution was done using 90% of solvent A and 10% of solvent B for one minute. After that a gradient elution from 90 to 10% solvent A and 10 to 90% of solvent B in twenty minutes was done, then elution with 90% of solvent B for four minutes was done then return to the initial condition (10% of acetonitrile) for three minutes. The flow rate was 0.3 mL/min. The instrument was coupled with Triple TOF 5600+ (Sciex^®^, Framingham, MA, USA) for IDA acquisition and Analyst TF 1.7.1 (Sciex^®^) for LC-Triple TOF control. Raw data files were loaded into MS-DIAL 3.52 for data-independent MS/MS deconvolution [69]. Compounds were recognized with >70% probability using an MS1 and MS2 tolerance of 0.2 mass units to be accepted as positive identifications. The ReSpect negative (1573 records) database was used as a reference database. PeakView 2.2 with the MasterView 1.1 package (AB SCIEX, Framingham, MA, USA) were used for feature or peaks extraction from the total ion chromatogram (TIC) based on that the signal-to-noise of features is more than ten, as well as their intensities of the sample-to blank should be more than three [37].

### 3.3. Bacteria

Twenty-three *S. aureus* isolates were from clinical specimens, including blood, wounds, sputum, and urine. They were identified by standard biochemical tests.

### 3.4. Antibiotic Susceptibility Testing

The antibiotic sensitivity of the *S. aureus* isolates was explored by the Kirby–Bauer disk diffusion technique. Mueller–Hinton agar (MHA) plates are utilized in this assay [70]. The following antibiotics were used: oxacillin (OX; 1 μg), erythromycin (E; 15 μg), gentamicin (GN; 10 μg), linezolid (LZD; 30 μg), clindamycin (DA; 2 μg), tetracycline (TE; 30 μg), cotrimoxazole (COT; 1.25/23.75 μg), minocycline (MI; 30 μg), gatifloxacin (GAT; 5 μg), chloramphenicol (C; 30 μg), azithromycin (AZM; 15 μg), and ciprofloxacin (CIP; 5 μg).

### 3.5. Antibacterial Action of C. droserifolia Methanol Extract

Antibacterial action was revealed by agar well diffusion in MHA plates [71]. The bacterial suspension (0.5 McFarland) was dispersed on the surface of the MHA plates. Three wells were performed. The first well-received CDE (2 mg/mL), the second received linezolid (positive control), and the third received dimethyl sulfoxide (DMSO, negative control). The appearance of inhibition zones revealed CDE’s antibacterial activity after incubating the plates at 37 °C for 24 h [72].

### 3.6. Determination of the Minimum Inhibitory Concentration (MIC) of CDE

The broth microdilution assay in MH broth was employed to estimate the MIC values of CDE against *S. aureus* isolates, as previously reported [72]. The MIC had the lowest concentration of CDE, and no growth was detected visually after overnight incubation at 37 °C [73].

### 3.7. Biofilm Inhibition

The effect of CDE on biofilm formation was investigated at 0.5 MIC values [74]. A tryptone soy broth (TSB) suspension was prepared from an over-night bacterial culture, and was adjusted to 10^6^ CFU/mL in freshly prepared TSB. Then, 200 µL of the bacterial suspension was added to the microtitration plates and wells in the presence and absence of sub-MIC (0.5 MIC) of SAM and incubated at 37 °C for 48 h. The TSB was gently removed, and the wells were washed to remove any planktonic cells and subsequently left for air drying. Add 200 µL of 99% methanol for 20 min, and then the formed biofilm was stained with 200 µL of 1% crystal violet (CV) solution for 15 min. After washing the plate, 33% glacial acetic acid was utilized as a solvent for CV. Using a microtitration plate reader (Sunrise, Männedorf, Switzerland), the absorbance of the solubilized dye was measured at 570 nm [72].

### 3.8. Scanning Electron Microscope (SEM)

The antibiofilm action of CDE on *S. aureus* bacteria was visualized under SEM, as previously explained (JEOL, Tokyo, Japan) [75].

### 3.9. Gene Expression Measurement Using qRT-PCR

The influence of CDE on the expression levels of the biofilm genes (*cna*, *fnb*A, and *ica*A) was elucidated using qRT-PCR. After growing the isolates in TSB in the presence and absence of sub-MICs of SAM, they were incubated overnight at 37 °C. After the incubation period, cells were harvested by centrifugation and immediately stored at −80 °C. The total RNA from *S. aureus* isolates was extracted and purified using TRIzol^®^ reagent (Life Technologies, Carlsbad, CA, USA) following the manufacturer protocol. Reverse transcription was employed using the QuantiTect Reverse Transcription kit (Qiagen, Hilden, Germany). Then, the formed cDNA was amplified using Maximas SYBR Green/Fluorescein qPCR master mix (Thermo Fisher Scientific, Waltham, MA, USA). The average threshold cycle (CT) values were normalized to the housekeeping gene (16s rRNA). The relative gene expression of the treated isolates was compared to that of the untreated ones according to the 2^−∆∆Ct^ method [76]. Primers are exposed in Table S1 [77,78].

### 3.10. In Vivo Assay

Forty male mice weighing 25–30 g and aged 6–8 weeks were obtained from the faculty of pharmacy at Tanta University, Egypt. They were grouped into four groups, each with ten mice. The first group was a normal control. The residual three groups were infected with 0.1 mL via intravenous injection of 1.5 × 10^7^ colony-forming units (CFUs) of *S. aureus* [79]. The second group served as a positive control group (placebo), and the third group administered linezolid (160 mg/kg/24 h) orally as a standard drug. The fourth group administered CDE orally (200 mg/kg/24 h) [12]. The experimental procedures were approved by the ethics committee at the faculty of pharmacy, Tanta University (TP/RE/3/24 p-03).

After two weeks, mice from the diverse groups were euthanized. Liver and spleen samples were obtained from each group. The bacterial burden was determined in the liver and spleen after homogenization. A 1:10 serial dilution of the tissue homogenates in phosphate buffered saline was performed. Then, 100 μL of undiluted and each subsequent dilution were spread onto tryptic soya agar plates in duplicate using a glass spreader. The plates were then incubated at 37 °C overnight.

The number of colonies was counted on each plate, and the count of CFU/mL was determined as follows: CFU/mL = number of colonies × dilution factor/0.1 mL. CFU/g = CFU/mL × number of mL/g [80].

On the other hand, tissue samples (2 × 3 mm) were excised, fixed in buffered formalin (10%), treated as previously described [81], and finally stained with hematoxylin and eosin (H&E) and photographed using a light microscope. Also, tumor necrosis factor-alpha (TNF-α) monoclonal antibodies were utilized for staining the tissues. The immunostained tissues were then checked by a light microscope.

### 3.11. ELISA

The anti-inflammatory potential of CDE was illuminated by determining the levels of the inflammatory mediators, interleukin IL-1β and IL-6 in pg/mg protein in the liver and spleen tissues by an ELISA kit from Abcam Co., Waltham, MA, USA, following the manufacturer’s instructions.

### 3.12. Histopathological Examination

Formalin-fixed hepatic and splenic tissues were processed, and 5-µm-thick paraffin sections were stained with hematoxylin and eosin (H&E). Photomicrographs were taken at different magnification powers using a light microscope (Olympus, Tokyo, Japan) to assess the morphological changes [82]. Histopathological evaluation of the hepatic and splenic tissue damage was performed. A score of zero indicates the absence of tissue necrosis; a score of one indicates mild damage, 10–20% liver cell degeneration, necrosis, and 10–20% red blood cell depletion. A score of two indicates moderate degeneration in the form of 20–40% liver cell degeneration, necrosis, and 20–40% red blood cell depletion in the spleen. A score of three indicates severe degeneration in the form of >40% liver cell degeneration and necrosis and >40% red blood cell depletion in the spleen.

### 3.13. Immunohistochemistry

Six-micrometer tissue sections from the liver and spleen were subjected to immunohistochemical staining; they were first dewaxed, rehydrated by a diminishing alcohol series, and treated with 10% hydrogen peroxide in methanol for ten minutes. Following this, the sections were microwaved for ten minutes in 0.01 M sodium citrate buffer (pH 6.0), allowed to cool at room temperature, and then repeatedly washed with PBS for five minutes. After washing, antigens were recovered by autoclaving in citrate buffer for 11 min. Next, slices were incubated with primary antibodies for a whole night at 4 °C. Then, the tissues were treated for 30 min at room temperature with 3, 3-diaminobenzidine and a rabbit polyclonal TNF-α antibody. The tissue sections were mounted for visibility, washed in xylene, and subtly counterstained with hematoxylin. Slides were examined under a light microscope at a magnification of ×400 [83]. Using image analysis tools (Image J, 1.46a, NIH, Bethesda, MD, USA), morphometric analysis was carried out. At ×400 magnification, the mean area percentage of TNF-α protein expression for each of the experimental groups was evaluated in ten non-overlapping fields within each region.

### 3.14. Statistics

The assays were carried out three times and exposed as mean ± standard deviation (SD). ANOVA was utilized to reveal the significance of differences among the experimental groups by GraphPad software version 8.0 (GraphPad Software, LLC, Boston, MA, USA). Results of the histopathological scores were analyzed using Kruskal–Wallis test followed by Dunn’s multiple comparison test.

## 4. Discussion

Medicinal plants can act as a natural source of numerous therapeutic compounds that can be utilized safely to treat various diseases in humans and animals [84]. In recent decades, increasing attention has been paid to elucidating plants as an important source for numerous drugs, principally antimicrobials, to combat multidrug-resistant bacteria [85]. Here, the tested *S. aureus* isolates were from blood (52.17%), wounds (21.73%), sputum (17.4%), and urine (8.7%). Previous studies have documented that most recovered *S. aureus* isolates were from blood and wounds [86,87,88]. Regarding the susceptibility to antibiotics, the isolates tested in our study revealed multidrug resistance comparable to previous reports [89,90,91].

CDE revealed antibacterial action on *S. aureus* isolates with MIC values of 128–512 µg/mL. An earlier study described the antibacterial action of CDE on *S. aureus* NCTC 10788, *Salmonella senftenberg* ATCC 8400, *Escherichia coli* BA 12296, and *Candida albicans* ATCC MAY-2876 [12]. In addition, the antibacterial action of the essential oil obtained from *Cleome* species was previously reported on Gram-positive and Gram-negative bacterial species [92].

The phytochemical analysis by LC-ESI-MS/MS of *C. droserifolia* methanol extract tentatively identified 44 compounds belonging to different entities. Flavonoids, anthocyanin, and organic acids composed a major part of the extract, and it was found that the major constituents were kaempferol-3,7-*O*-bis-alpha-L-rhamnoside, isorhamnetin, cyanidin-3-glucoside, 3, 5, 7-trihydroxy-4′-methoxyflavone (kaempferide), kaempferol-3-*O*-alpha-L-rhamnoside, caffeic acid, isoquercitrin, quinic acid, isocitrate, mannitol, apigenin, acacetin, and naringenin. Kaempferol glycosides were reported to exert antimicrobial and anti-inflammatory effects [93,94]. Isorhamnetin, or 3′-methoxylated quercetin derivative, is a flavanol with antidiabetic, anti-inflammatory, and antimicrobial effects [95]. Flavonoids such as kaempferol derivatives, isorhamnetin, apigenin, acacetin, and naringenin are valuable groups of compounds with antimicrobial and anti-biofilm activities [96].

Wang et al. reported that cyanidin-3-glucoside exhibited antimicrobial and anti-inflammatory potential [97]. Cyanidin-3-glucoside inhibits the NF-κB pathway. Also, it was reported to inhibit the interferon-mediating inflammatory cascades and reduce the proinflammatory cytokines, such as interferon-γ, TNF-α, interleukin (IL)-5, IL-9, and IL-10 [95,98].

As biofilm is an important virulence factor for *S. aureus*, it enables it to resist multiple antibiotics by transferring the genes of resistance among the bacterial cells embedded in the biofilms and by hindering the penetration of the antibiotics across the biofilm [99,100]. Thus, antibiofilm agents are beneficial for managing MDR *S. aureus* infections [101]. Herein, CDE revealed antibiofilm action by crystal violet and SEM. Also, it revealed a downregulating effect on the biofilm-encoding genes (*cna*, *fnb*A, and *ica*A) using qRT-PCR in 43.48% of the isolates. Such genes encode intracellular adhesion molecules (*ica*A) and microbial surface components recognizing adhesive matrix molecules (*fnb*A and *cna*), which have a great role in biofilm formation [102].

Regarding the *in vivo* model, which was employed to simulate the human body [103], CDE revealed a promising effect in the studied infection model as it significantly decreased the bacterial count in the liver and spleen, indicating its antibacterial action *in vivo*. Also, it improved the histological features of the liver and spleen, manifested by regaining the normal hepatic structure and the splenic architecture.

As bacterial infections are among the causes that often trigger inflammation as a body response [104], we elucidated the consequence of CDE on the inflammatory markers in the liver and spleen using immunohistochemistry and ELISA. TNF-α is an inflammatory cytokine produced by macrophages as a response to acute inflammation [105,106]. Also, IL-1β is a proinflammatory cytokine that mediates numerous physiological responses, such as fever and lymphocyte activation [107,108]. IL-6 is an important pleiotropic cytokine in the inflammatory response [109,110,111]. Remarkably, CDE has significantly diminished these inflammatory mediators, which could have a role in its antibacterial potential. The experiment demonstrated a notable enhancement in the survival rate of rats infected with *S. aureus* when treated with CDE compared to the positive control group. CDE efficacy was comparable to that of the standard drug, linezolid. These results highlight the potential therapeutic value of CDE as an alternative treatment for *S. aureus* infections, warranting further investigation into the clinical relevance of these findings.

## 5. Conclusions

LC-ESI-MS/MS revealed that *C. droserifolia* methanol extract had variable phytochemicals with valuable biological activities. Flavonoid glycosides, anthocyanins, and other phenolic compounds were believed to cause multiple effects, such as antimicrobial, antibiofilm, and anti-inflammatory properties. The predominant compounds in CDE were kaempferol-3,7-*O*-bis-alpha-L-rhamnoside, isorhamnetin, cyanidin-3-glucoside, 3, 5, 7-trihydroxy-4′-methoxyflavone (kaempferide), kaempferol-3-*O*-alpha-L-rhamnoside, caffeic acid, isoquercitrin, quinic acid, isocitrate, mannitol, apigenin, acacetin, and naringenin. CDE exhibited a potent antibacterial action on *S. aureus* isolates with MICs that ranged from 128 to 512 µg/mL. It also revealed antibiofilm action using a crystal violet assay and SEM. This antibiofilm potential was further studied at the molecular level using qRT-PCR on the biofilm-encoding genes, and it revealed a downregulating action on the studied genes in 43.48% of the isolates. In the *in vivo* aspect, using ELISA and immunohistochemical studies, the CDE-treated group showed a significant improvement in the histological features, with a significant lessening in the inflammatory markers in the liver and spleen. Our work has shown that treatment with CDE can significantly improve the survival rate of *S. aureus*-infected rats, with efficacy similar to that of the standard drug linezolid, suggesting potential therapeutic value and prompting further exploration of its clinical relevance. Thus, it is important to perform future studies on CDE to reveal its potential activity on other bacterial species and to elucidate its action in clinical practice.

## Figures and Tables

**Table 1:** The phytoconstituents of *C. droserifolia* methanol extract recognized by LC-ESI-MS/MS analysis.

	RT (min)	Compound Name	Precursor *m/z*	Error ppm	Formula	MS/MS	Ontology	Reference
1	0.821	Succinic acid	117.0192 [M − H]^−^	−0.2	C_4_H_6_O_4_	73.21, 100.00, 117.07	Dicarboxylic acids and derivatives	[30]
2	0.821	3-Hydroxy-3-Methylglutaric acid (Meglutol)	161.0471 [M − H]^−^	−7.8	C_6_H_10_O_5_	56.85, 113.05, 131.03, 143.04, 161.02	Hydroxy fatty acids	[31]
3	0.860	cis-Aconitate	173.0816 [M − H]^−^	0.4	C_6_H_6_O_6_	111.43, 129.12, 173.02	Tricarboxylic acids derivatives	[32]
4	0.912	D-(-)-Quinic acid	191.0553 [M − H]^−^	3.2	C_7_H_12_O_6_	111.02, 155.02, 164.02, 173.02, 191.77	Quinic acids and derivatives	[33]
5	0.939	Catechol	109.0291 [M − H]^−^	0.8	C_6_H_6_O_2_	53.03, 65.00, 81.03, 91.02, 109.02	Catechols	[34]
6	0.940	3,4-dihydroxy benzoic acid	153.0191 [M − H]^−^	0.1	C_7_H_6_O_4_	53.03, 79.95, 108.12, 153.01	Hydroxybenzoic acid derivatives	[35]
7	9.581	3, 5, 7-trihydroxy-4′-methoxyflavone (kaempferide)	299.0563 [M − H]^−^	0.2	C_16_H_12_O_6_	64.02, 107.00, 151.09, 164.12, 253.63, 284.53, 299.01	Flavonols	[36]
8	1.006	Maleic acid	115.0388 [M − H]^−^	7.4	C_4_H_4_O_4_	69.03, 71.04, 115.04	Dicarboxylic acids and derivatives	[37]
9	1.256	Isorhamnetin	315.0754 [M − H]^−^	−8.9	C_16_H_12_O_7_	56.01, 135.02, 109.03, 151.04, 163.04,255.42, 271.12, 300.23, 315.16	Flavonol	[38]
10	1.138	Mannitol	181.0728 [M − H]^−^	−4.4	C_6_H_14_O_6_	59.02, 71.32, 89. 14, 101.05, 163.05, 181.99	Sugar alcohols	[39]
11	1.152	Caffeic acid	179.0567 [M − H]^−^	−2.2	C_9_H_8_O_4_	77.70, 89.03,117.10, 134.09, 161.05, 179.06	Hydroxycinnamic acids	[40]
12	3.321	p-Coumaric acid	163.0401 [M − H]^−^	−0.7	C_9_H_8_O_3_	65.97, 91.03, 107.03, 119.04, 163.04	Hydroxycinnamic acids	[41]
13	3.346	(R)-2-hydroxy-3-butenyl glucosinolate	388.0759 [M − H]^−^	−3.4	C_11_H_19_NO_10_S_2_	195.02, 241.14, 259.00, 290.97, 388.03	Alkyl glucosinolates	[42]
14	4.432	Esculin	339.0709 [M − H]^−^	0.4	C_15_H_16_O_9_	69.01, 121.09, 178.84, 320.10, 339.34	Coumarin glycosidesNIST	[43]
15	5.318	Chlorogenic acid	353.0841 [M − H]^−^	7.2	C_16_H_18_O_9_	135.02, 161.02, 179.11, 191.06, 353.20	Quinic acids and derivatives	[44]
16	5.460	Isoquercitrin	463.0854 257; 229; 201; 150; 155	4.6	C_21_H_20_O_12_	65.00, 150.99, 229.95, 257.04, 463.04	Flavonoid-3-*O*-glycosides	[45]
17	5.5115	Luteolin	285.1688 [M − H]^−^	4.4	C_15_H_10_O_6_	216.98, 199.04, 175.04, 151.01, 285.03	Flavones	[46]
18	5.798	Kaempferol-3-*O*-(6-p-coumaroyl)-glucoside	593.1558 [M − H]^−^	−6.5	C_30_H_26_O_13_	56.04, 447.11, 430.91, 307.21, 285.18	Flavonoid 3-*O*-p-coumaroyl glycosides	[46]
19	5.987	Syringetin-3-*O*-glucoside	507.1159 [M − H]^−^	−2.2	C_23_H_24_O_13_	112.98, 302.89, 329.94, 345.08, 507.01	Flavonoid-3-*O*-glycosides	[47]
20	6.101	Delphinidin-3-*O*-β-glucopyranoside (Myrtillin A)	463.0253 [M − 2H]^−^	−1.7	C_21_H_21_O_12_	125.02, 271.018, 300.05, 301.02, 463.03	Anthocyanidin-3-*O*-glycosides	[48]
21	6.254	Cyanidin-3-glucoside	447.0908 [M − H]^−^	4.4	C_21_H_21_O_11_	147.05, 227.00, 256.20, 285.42, 447.07	Anthocyanidin-3-*O*-glycosides	[48,49]
22	6.329	Luteolin-3′, 7-di-*O*-glucoside	609.1434 [M − H]^−^	3.2	C_27_H_30_O_16_	112.97, 253.00, 285.14, 399.05, 447.144, 489.02, 609.13	Flavonoid-7-*O*-glycosides	[50]
23	6.374	3-(4-hydroxy-3,5- dimethoxyphenol)-2-propenoic acid sinapic acid or sinapinic acid	223.0618 [M − H]^−^	−2	C_11_H_12_O_5_	59.01, 175.03, 207.04, 223.13	Hydroxycinnamic acids	[51]
24	6.601	Quercitrin	447.1836 [M − H]^−^	3.9	C_21_H_20_O_11_	152.11, 300.18, 301.12, 447.12	Flavonoid-3-*O*-glycosides	[52]
25		quercetin-3′-methoxy-3*O*-(4″-acetylrhamnoside)-7-*O*-rhamnoside	665.165 [M − H]^−^		C_30_H_50_O_16_	315.16, 461.01, 519.32, 665.21	Flavonoid-3-*O*-glycosides	[53]
26	6.614	Isocitrate	191.0334 [M − H]^−^	4.3	C_6_H_8_O_7_	76.03, 107.01, 149.02, 191.02	Tricarboxylic acids and derivatives	[54]
27	6.764	Benzyl glucosinolate	407.0401 [M − H]^−^	−0.7	C_14_H_18_NO_9_S_2_	212.03, 240.99, 259.00, 274.01, 328.07, 407.04	Hydroxy cinnamic acids	[42,55]
28	6.891	Kaempferol-3-*O*-α-L-rhamnoside	431.099 [M − H]^−^	−0.7	C_21_H_20_O_10_	89.05, 285.13, 313.12, 395.03, 430.96, 431.45	Flavonoid-3-*O*-glycosides	[56]
29	7.013	Isorhamnetin-3-*O*-rutinoside	623.163 [M − H]^−^	−1.1	C_28_H_32_O_16_	151.00, 165.01, 315.23, 623.72	Flavonoid-3-*O*-glycosides	[57]
30	7.025	Peonidin-3,5-*O*-di- β-glucopyranoside	623.0959 [M − 2H]^−^	−3	C_28_H_33_O_16_	59.01, 301.01, 463.11, 623.16	Anthocyanidin-5-*O*-glycosides	[58]
31	7.062	Delphinidin-3-*O*-(6″-*O*-alpha-rhamnopyranosyl-beta-glucopyranoside)	609.2933 [M − 2H]^−^	−7.1	C_27_H_31_O_16_	125.01, 300.03, 301.01, 447.16, 462.91, 609.13	Anthocyanidin-3-*O*-glycosides	[46,48]
32	7.515	Kaempferol-3,7-*O*-bis-α-L-rhamnoside	577.1559 [M − H]^−^	0.7	C_27_H_30_O_14_	285.0405, 431.0969, 577.43	Flavonoid-7-*O*-glycosides	[46]
33	7.552	Kaempferol-3-*O*-α-L-arabinoside	417.0826 [M − H]^−^	−1.9	C_20_H_18_O_10_	258.43, 285.32, 313.02, 417.33	Flavonoid-3-*O*-glycosides	[59]
34		kaempferol-4′-methoxy-3,7- dirhamnoside	591.24 [M − H]^−^		C_28_H_32_O_14_	285.02, 299.21, 445.02, 591.11	Flavonoid-3-*O*-glycosides	[60]
35		isorhamnetin-3-*O*-β-d-glucoside	477.23 [M − H]^−^			269.98, 300.52, 315.03, 477.03	Flavonoid-3-*O*-glycosides	[59,61]
36	7.564	Hesperetin	301.1482 174.92, 255.22, 301.06 [M − 2H]^−^	−2.9	C_16_H_14_O_6_	153.10, 177.61, 273.13, 273.06, 301.00	4′-*O*-methylated flavonoids	[62]
37	7.626	Apigenin	269.1399 [M − H]^−^	0.3	C_15_H_10_O_5_	149.01, 151.09, 183.12, 225.05, 241.00, 269.06	Flavones	[63]
38	7.851	Naringenin	271.0617 [M − H]^−^	0.7	C_15_H_12_O_5_	63.02, 151.00, 177. 227.08, 227.06, 271.06	Flavanones	[64]
39	8.009	apigenin-7-*O*-glucoside	431.0987 [M − H]^−^	−0.6	C_21_H_20_O_10_	268.14, 269.09, 310.88, 431.02	Flavonoid-7-*O*-glycosides	[65]
40	8.136	Peonidine-3-*O*-glucoside	461.1121 [M − 2H]^−^	−4.2	C_22_H_23_O_11_	71.01, 79.05, 89.02, 301.98, 461.30	Anthocyanidin-3-*O*-glycosides	[48,58]
41	8.274	Daidzein-8-C-glucoside	415.1986 [M − H]^−^	−1.2	C_21_H_20_O_9_	249.14, 267.03, 295.16, 325.20, 379.23, 415.87	Isoflavonoid C-glycosides	[66,67]
42	8.999	Syringaldehyde	181.051 [M − H]^−^	0.8	C_9_H_10_O_4_	99.97, 136.00, 151.02, 166.04 181.09	Methoxy phenols	[36]
43	10.301	Maritimetin-6-*O*-glucoside	447.2719 [M − H]^−^	1.8	C_21_H_20_O_11_	57.03, 132.71, 151.03, 285.05, 447.32	Aurone O-glycosides	[46]
44	13.88	Acacetin	283.0594 [M − H]^−^	5.1	C_16_H_12_O_5_	151.08, 252.13, 240.04, 268.03, 283.26	4′-*O*-methylated flavonoids	[63,68]

**Table 2:** Influence of CDE on the biofilm-forming ability of *S. aureus* isolates.

Biofilm Forming Ability *	Number of Isolates	
	Before Treatment	After Treatment
Non-biofilm forming (NBF)	1	5
Weak biofilm forming (WBF)	5	11
Moderate biofilm forming (MBF)	8	3
Strong biofilm forming (SBF)	9	4

* The tested isolates were characterized into four groups based on their ODs as follows: i. NBF: ODc < OD < 2 ODc. ii. WBF: 2 ODc < OD < 4 ODc. iii. MBPF: 4 ODc < OD < 6 ODc. iv. SBF: 6 ODc < OD. The cut-of OD (ODc) is the mean OD plus 3 SD of the negative control.

**Table 3:** Effect of CDE on IL-1β and IL-6 levels in the liver and spleen of the experimental groups.

	Inflammatory Levels (pg/mg Protein)			
Groups	Liver		Spleen	
	IL-1β	IL-6	IL-1β	IL-6
Group I	12.3 ± 2.3	19.8 ± 3.2	13.3 ± 0.8	24.3 ± 2.5
Group II	80.5 ± 3.5 *	219.2 ± 14.5 *	89.3 ± 7.4 *	240.6 ± 18.4 *
Group III	19.4 ± 1.4	28.9 ± 4.2	20.2 ± 2.3	34.6 ± 4.7
Group IV	22.7 ± 1.1	29.3 ± 5.3	24.3 ± 1.4	36.9 ± 5.8

The symbol (*) denotes a significant difference (*p* < 0.05).

## Data Availability

Data are contained within the article and Supplementary Materials.

## Supplementary Materials

The following supporting information can be downloaded at: https://www.mdpi.com/article/10.3390/antibiotics13050450/s1, Table S1. Sequences of the utilized primers. Table S2. Inhibition zone diameter of SAM, linezolid (positive control), and DMSA (negative control). Table S3. Minimum inhibitory concentrations (MICs) of SAM. Figure S1. Negative mode total ion chromatogram of LC-ESI-MS/MS of *Cleome droserifolia* methanol extract.
