# Supplementary material for: Potential Surviving Effect of Cleome droserifolia Extract against Systemic Staphylococcus aureus Infection: Investigation of the Chemical Content of the Plant
Source: Antibiotics (Basel). 2024 May 15;13(5):450. doi: 10.3390/antibiotics13050450 (PMC11118852; doi:10.3390/antibiotics13050450)
Supplement: Supplementary file 1 [file antibiotics-13-00450-s001.zip › antibiotics-2998919-supplementary.pdf]

Potential Surviving Effect of *Cleome droserifolia* Extract against Systemic *Staphylococcus aureus* Infection: Investigation of the Chemical Content of the Plant

Table S1. Sequences of the utilized primers

Gene	Forward Primer (5′ to 3′)	Reverse Primer (5′ to 3′)
*ica*A (intercellular adhesion gene)	GAGGTAAAGCCAACGCACTC	CCTGTAACCGCACCAAGTTT
*fnb*A (fibronectin-binding protein A)	AAATTGGGAGCAGCATCAGT	GCAGCTGAATTCCCATTTTC
cna (collagen binding protein)	AATAGAGGCGCCACGACCGT	GTGCCTTCCCAAACCTTTTGAGC
16S rRNA (housekeeping gene)	GGGACCCGCACAAGCGGTGG	GGGTTGCGCTCGTTGCGGGA

Figure S1. Negative mode total ion chromatogram of LC-ESI-MS/MS of *Cleome droserifolia* methanol extract.

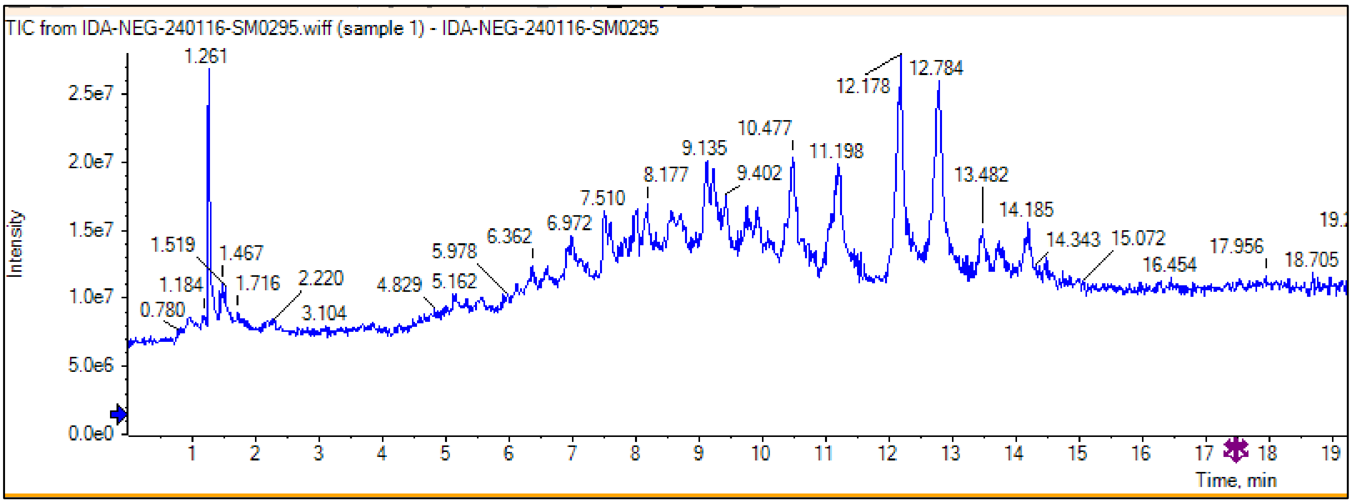

Table S2. Inhibition zone diameter of SAM, linezolid (positive control), and DMSA (negative control).

**Isolates**	**Inhibition zone diameter (mm)**		
	**SAM**	**Linezolid**	**DMSO**
**S1**	21	25	0
**S2**	20	23	0
**S3**	22	25	0
**S4**	23	26	0
**S5**	24	22	0
**S6**	25	30	0
**S7**	21	27	0
**S8**	22	28	0
**S9**	23	26	0
**S10**	24	28	0
**S11**	23	25	0
**S12**	24	26	0
**S13**	23	23	0
**S14**	23	27	0
**S15**	23	25	0
**S16**	24	28	0
**S17**	25	23	0
**S18**	23	22	0
**S19**	21	23	0
**S20**	22	24	0
**S21**	23	24	0
**S22**	23	25	0
**S23**	20	26	0

Table S3. Minimum inhibitory concentrations (MICs) of SAM.

Isolate code	MIC (µg/mL)	Isolate code	MIC (µg/mL)
S1	128	S13	128
S2	128	S14	256
S3	256	S15	128
S4	128	S16	512
S5	512	S17	512
S6	512	S18	256
S7	128	S19	128
S8	128	S20	128
S9	512	S21	512
S10	256	S22	256
S11	256	S23	128
S12	512		
